# Sanguinarine Inhibition of TNF-α-Induced CCL2, IKBKE/NF-κB/ERK1/2 Signaling Pathway, and Cell Migration in Human Triple-Negative Breast Cancer Cells

**DOI:** 10.3390/ijms23158329

**Published:** 2022-07-28

**Authors:** Samia S. Messeha, Najla O. Zarmouh, Lovely Antonie, Karam F. A. Soliman

**Affiliations:** 1 Division of Pharmaceutical Sciences, College of Pharmacy & Pharmaceutical Sciences, Institute of Public Health, Florida A&M University, Tallahassee, FL 32307, USA; samia.messeha@famu.edu (S.S.M.); lovely1.antonie@famu.edu (L.A.); 2 Faculty of Medical Technology-Misrata, Libyan Ministry of Technical & Vocational Education, Misrata LY72, Libya; najlazar@yahoo.com

**Keywords:** sanguinarine, triple-negative breast cancer, MDA-MB-231, MDA-MB-468, inflammatory angiogenesis, migration, cytokine

## Abstract

Angiogenesis is a process that drives breast cancer (BC) progression and metastasis, which is linked to the altered inflammatory process, particularly in triple-negative breast cancer (TNBC). In targeting inflammatory angiogenesis, natural compounds are a promising option for managing BC. Thus, this study was designed to determine the natural alkaloid sanguinarine (SANG) potential for its antiangiogenic and antimetastatic properties in triple-negative breast cancer (TNBC) cells. The cytotoxic effect of SANG was examined in MDA-MB-231 and MDA-MB-468 cell models at a low molecular level. In this study, SANG remarkably inhibited the inflammatory mediator chemokine CCL2 in MDA-MB-231 and MDA-MB-468 cells. Furthermore, qRT-PCR confirmed with Western analysis studies showed that mRNA CCL2 repression was concurrent with reducing its main regulator IKBKE and NF-κB signaling pathway proteins in both TNBC cell lines. The total ERK1/2 protein was inhibited in the more responsive MDA-MB-231 cells. SANG exhibited a higher potential to inhibit cell migration in MDA-MB-231 cells compared to MDA-MB-468 cells. Data obtained in this study suggest a unique antiangiogenic and antimetastatic effect of SANG in the MDA-MB-231 cell model. These effects are related to the compound’s ability to inhibit the angiogenic CCL2 and impact the ERK1/2 pathway. Therefore, SANG use may be recommended as a component of the therapeutic strategy for TNBC.

## 1. Introduction

Cancer cells are characterized by an increased metabolic rate that requires a sufficient blood circulation system for providing nutrients, supplying oxygen, and eliminating their byproduct waste. These requirements are provided by inducing new blood vessels in the tumor by utilizing the angiogenesis mechanism [1,2]. Indeed, tumor angiogenesis is evidenced by higher vascularization in rapidly growing cancer types compared with dormant cancer and is considered crucial in tumor growth [2,3]. This is also applied to breast cancer (BC), the heterogeneous disease with aggressive subtypes such as triple-negative breast cancer (TNBC), with complicated molecular, dynamic, morphological, and clinical outcomes [4,5,6,7]. The treatment of TNBC is challenging due to the absence of three particular receptors: estrogen (ER), progesterone (PR), and human epidermal growth factor (Her2/neu) [7,8,9]. Even though a substantial initial response to various chemotherapy agents was found, around 30% of TNBC patients experience treatment failure after frequent exposure to this treatment [7,10]. One of the hallmarks of cancer is angiogenesis, where new blood supplies will sustain tumor development, proliferation, and metastasis [11,12,13].

The process of tumor angiogenesis and neovascularization are synchronized with the progression of BC [14,15]. Consequently, the level of angiogenesis is considered one of the prognostic markers for survival in BC patients [16,17]. In malignant tumors, the typical angiogenic process is initiated by releasing angiogenic factors into the extracellular media, stimulating endothelial cell proliferation [18,19,20,21,22,23,24,25]. Balanced pro- and anti-angiogenic factors are crucial to maintaining vascular homeostasis by preventing endothelial cell proliferation and keeping vasculature quiescent. The unregulated cell signal and cell mutation in the TNBC could lead to persisting proliferative signaling and angiogenesis, prompting invasion and metastasis [26]. In the tumor microenvironment, however, the pro-angiogenic signaling is dominating, leading to deregulated tumor angiogenesis [3,22], known as the “angiogenic switch” [27]. This mechanism can be provoked through various manifestations, including increased proliferation, genetic alteration of cancer cells, and tumor-related inflammation [3,22]. More importantly, however, is the upregulated expressions of angiogenic growth factors, which reflect the invasive BC [27,28,29]. Nevertheless, altered inflammatory pathways associated with chronic inflammation lead to pathological angiogenesis [18]. These relationships are evidenced in the advanced stages of cancer and pioneer the concept of angiogenesis-cancer-associated death [30]. Hence, a precisely controlled network of angiogenesis-linked inflammation is essential to sustain physiological homeostasis. Endothelial cells and macrophages produce angiostatic agents, including anti-inflammatory chemokines, to regulate tumor angiogenesis and inflammation [19,20,21,31,32,33,34,35,36,37]. Generally, these chemokines direct the migration and recruitment of macrophage cells to sites of inflammation [38,39,40]. Some chemokines are normally found under homoeostatic circumstances. Meanwhile, other CC-chemokines, such as CCL2, are expressed when inflammatory stimuli are triggered [41,42]. Increasing evidence emphasized the role of the CC-chemokines as a substantial regulator of angiogenesis, in addition to their distinct role in sustaining immune cell homeostasis [30,43,44,45,46]. The CC-chemokines have been demonstrated to activate NF-KB and ERK1/2 signaling pathways, which augment the process of neovascularization [43,47], inflammation-induced angiogenesis [48,49], migration [43,47,50,51,52,53], and chemotherapy/endocrine resistance [54]. Thus, exploring the potential to inhibit inflammatory angiogenesis and metastasis in TNBC cells becomes crucial.

On the other hand, typical anti-angiogenic therapies have emerged as a promising avenue in controlling disease progression. Unfortunately, available medications are frequently high in cost and linked to other side effects, such as high blood pressure and digestive system disorders [55,56]. This limitation emphasizes the demand for novel therapeutic strategies to significantly inhibit inflammation-stimulated angiogenesis, while protecting other vital physiological ischemia-controlling angiogenic mechanisms [57]. The notion that cancer is closely associated with uncontrolled angiogenesis prompted the utilization of plant-based compounds for angiogenesis inhibition in cancer [58]. Indeed, naturally derived compounds have been recommended in treating cancer because of their relative safety and low cost [59].

The benzophenanthridine alkaloid Sanguinarine (SANG), extracted from the root of *Sanguinaria canadensis* as well as other poppy Fumaria species, has been demonstrated to induce various biological activities, such as antioxidant, anti-inflammatory, and antimicrobial properties [60]. Additionally, in both in vitro and in vivo, SANG consistently showed safe effects [61]. In many preclinical studies, SANG at micromolar concentrations exhibited anticancer properties by inhibiting cell proliferation, invasion, and migration, and repressed angiogenesis [62,63,64,65,66,67,68]. The antiangiogenic effect of SANG has been validated in many cancer cells, including BC [63,66,67,69,70]. This property was suggested to be through blocking VEGF-stimulated vessel growth, as it reduced cell survival and migration [66,70,71,72,73]. Nevertheless, the potential of SANG to inhibit inflammatory angiogenesis has been meagerly studied in TNBC cells.

The current study is designed to explore the potential of the natural alkaloid SANG to inhibit inflammatory angiogenesis and metastasis factors in TNBC cell lines—using MDA-MB-231 and MDA-MB-468 cells—originating from Caucasian American (CA) and African American (AA) women, respectively. The mechanism mediating the anticancer activities was also explored by studying the impact of SANG on different signaling pathways implicated in angiogenesis.

## 2. Results

### 2.1. SANG Decreases the Viability of TNF-α-Treated TNBC Cells

**Figure 1:**
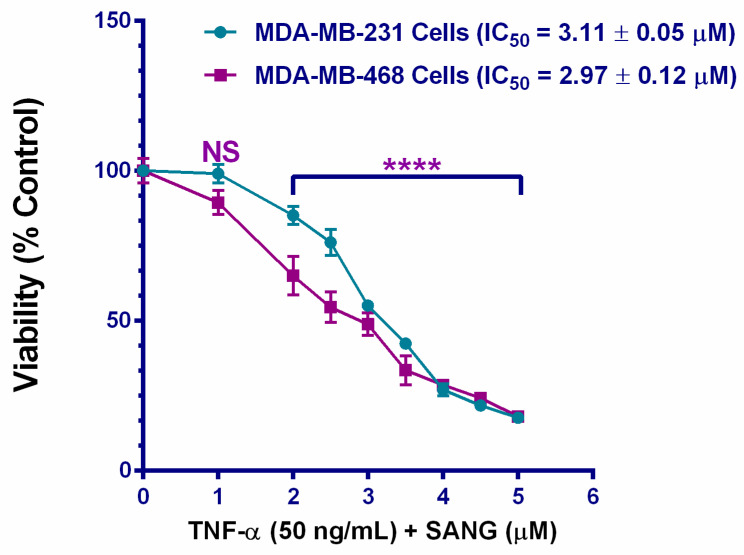
The cytotoxic effect of SANG in TNF-α-activated triple-negative breast cancer (TNBC) cells. MDA-MB-231 and MDA-MB-468 cell models were co-treated with 50 ng/mL TNF-α and SANG (0–5 µM) for 24 h. On the *x*-axis, the pointing arrows correspond to the designated concentrations to be applied for the following angiogenesis arrays. Each data point represents the mean ± SEM of three independent studies/n = 6 each. The percentages of cell survival were normalized to the DMSO-treated control cells, and IC50 s values were calculated using Prism software. One-way ANOVA followed by Bonferroni, a multiple comparison test, were employed to verify the statistical difference between the control and co-treated groups. **** *p* < 0.0001 is highly significant; NS is non-significant.

The cytotoxic effects of SANG were determined in TNF-α-activated TNBC cells by investigating the viability of TNF-α-treated MDA-MB-231 and MDA-MB-468 cells in the presence of gradual concentrations of SANG simultaneously (Figure 1). The reduction in resazurin, compared to the DMSO-treated control cells, indicated affected metabolic activities in the two treated cell lines. Following a 24 h exposure period, both cell models showed a highly significant response to the compound (*p* < 0.0001) at 2.0–5.0 µM, with non-significant responses at 1 µM. The IC50 values generated from the dose—response data analysis (IC50 = 3.11 ± 0.05 µM for MDA-MB-231 cells and 2.97 ± 0.12 µM for MDA-MB-468 cells) revealed similar inhibition potencies of SANG over these two models.

### 2.2. SANG Inhibits the Release of CCL2 Cytokine in TNBC Cells

**Figure 2:**
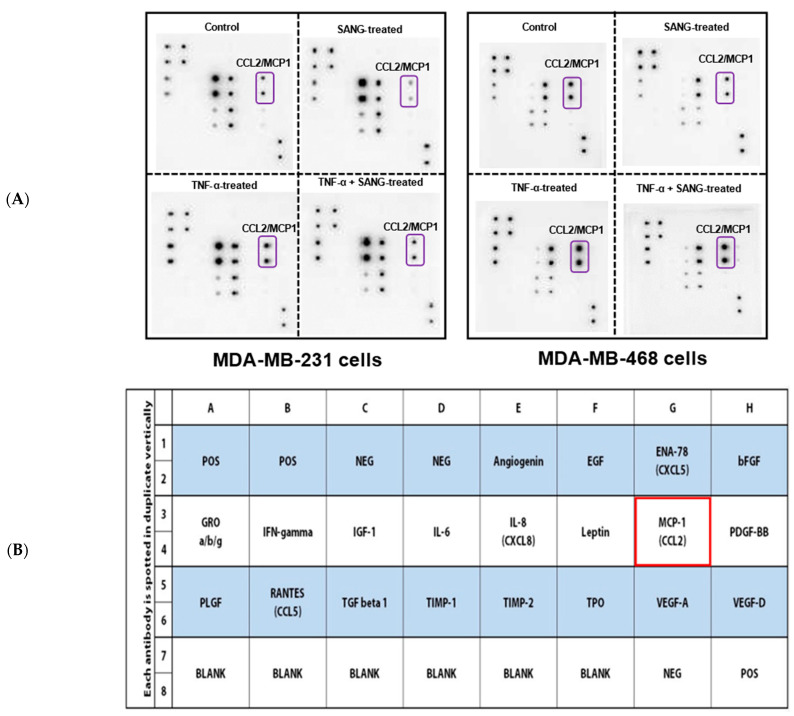

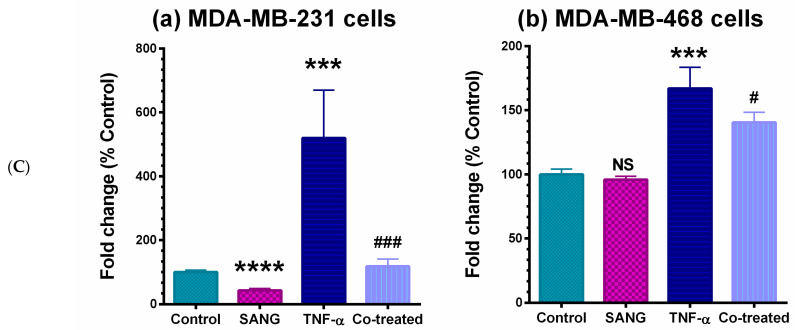
The inflammation-related antiangiogenic effects of SANG in TNF-α-stimulated TNBC cells. (**A**) The human angiogenesis microarray blots were used to measure the expression of various angiogenic chemokine/cytokine in the cell-free supernatant. For each cell line, the four blots correspond to control, SANG-treated, TNF-α-stimulated, and co-treated (TNF-α + SANG) cells. (**B**) The captured images revealed the altered expressions in the chemokine CCL2. The human angiogenesis microarray map shows the location of the impacted chemokine MCP1/CCL2 as denoted by a red frame. (**C**) Quantification of extracellular CCL2 released by differently treated TNBC cells. The significant difference between TNF α-treated cells vs. control cells (*) or between TNF-α-stimulated vs. co-treated (#) groups was determined using an unpaired *t*-test. **** *p* < 0.0001; ***/### *p* < 0.001; # *p* < 0.05; NS, not significant.

The inflammation-related antiangiogenic effect of SANG in TNBC-stimulated cells was established using the Human Angiogenesis microarray (Figure 2A–C). The levels of cytokines released by differently treated groups were visualized as spots with different intensities on the acquired images (Figure 2A). Blots of TNF-α-stimulated cell supernatant revealed the highest spot intensities. The expression of CCL2 was remarkably altered in both cell lines (framed with red microarray map Figure 2B), while non-significant changes were measured for other cytokines (data not presented). Translating spot intensities to numbers using angiogenesis data analysis software (Ray Biotech, Norcross, GA, USA) confirmed our visual observation (Figure 2A). The semiquantitative normalized data showed that CCL2 was significantly augmented (*p* < 0.001) in both TNF-α-stimulated TNBC cell lines (Figure 2C). Additionally, TNF-α stimulation of CCL2 was considerably higher in MDA-MB-231 cells than in its counterpart MDA-MB-468 cells (~4.5-fold vs. 1.5-fold increase, *p* < 0.001). In the presence of TNF-α, SANG dramatically attenuated the expression of CCL2 by more than 70% (*p* < 0.001) in MDA-MB-231 cells compared to only 15% (*p* < 0.05) in MDA-MB-468 cells. In other words, SANG was >4.5-fold more effective at lowering CCL2 in MDA-MB-231 cells than in MDA-MB-468 cells. Interestingly, the significant inhibition (~50% inhibition, *p* < 0.0001) of CCL2 was exclusively exhibited in SANG-treated MDA-MB-231 cells compared with the control.

**Figure 3:**
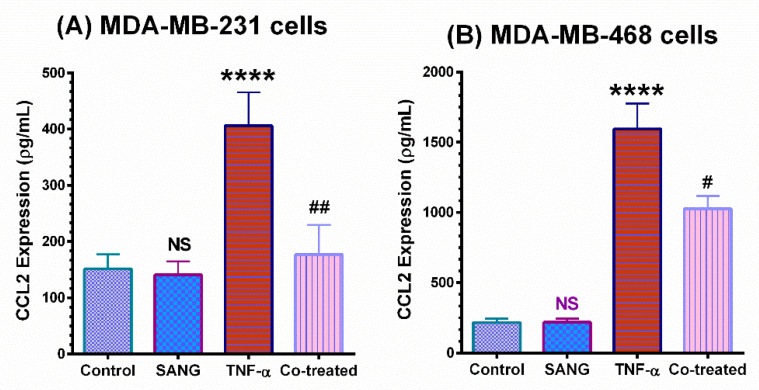
ELISA quantifications and validation for CCL2 release from (**A**) TNF-α-stimulated MDA-MB 231 and (**B**) MDA-MB-468 cells. The normalized data shows CCL2 expression (pg/mL) in four samples of cell supernatants, namely, resting, SANG-treated, TNF-α-treated cells, and co-treated cells (TNF-α + SANG). The data generated from three independent experiments are presented as the mean ± SEM. The significant difference between TNF α-treated cells vs. control cells (*) or between TNF-α-stimulated vs. co-treated cells (#) groups was analyzed using an unpaired *t*-test. **** *p* < 0.0001, ## *p* < 0.01, # *p* < 0.05 are considered significant difference; NS, not significant.

ELISA studies were performed to quantify and validate CCL2 protein expression (pg/mL) in the supernatant of each sample (Figure 3A,B). In both cell lines, the obtained ELISA data validated the blot findings as the results were consistent. In TNF-α-stimulated MDA-MB-231 cells, the significantly upregulated (*p* < 0.0001) angiogenic cytokine CCL2 was attenuated by 60% (*p* < 0.01), compared to ~40% (*p* < 0.05) inhibition in MDA-MB-468 cells.

### 2.3. SANG Represses the Expression of the IKBKE Gene Promoting CCL2 Release in Stimulated TNBC Cells

**Figure 4:**
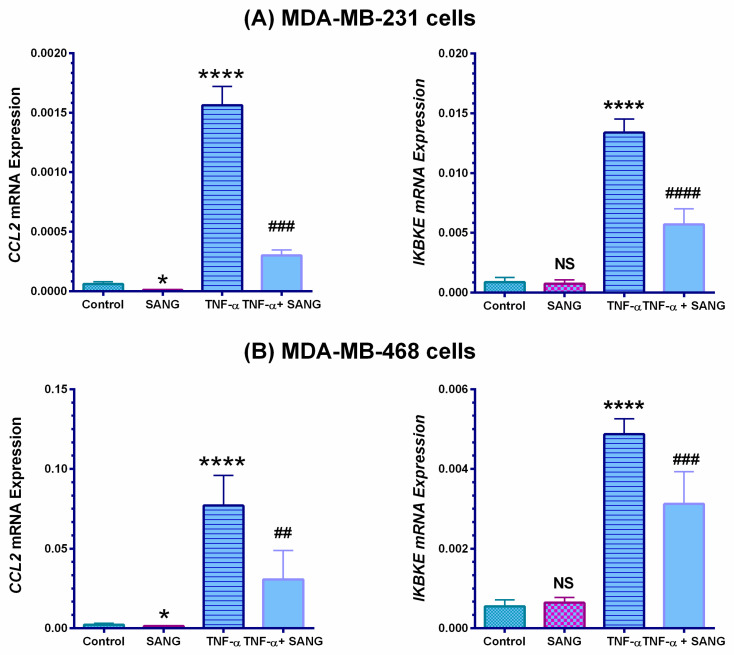
Effect of SANG on the angiogenic cytokine-related gene in TNF-α-stimulated MDA-MB-231 (**A**) and MDA-MB-468 (**B**) cells. GAPDH normalized PCR data revealed a significant upregulation in CCL2 and its regulator gene IKBKE mRNA. These genes were significantly repressed in the presence of 2 µM or 1.5 µM SANG in MDA-MB-231 and MDA-MB-468 cells, respectively. The data are presented as the mean ± SEM of three independent studies. An unpaired *t*-test was used to analyze the significance of the difference between TNF α-stimulated vs. < 0.1 DMSO-treated resting cells (*) and TNF-α-activated vs. co-treated cells (#). ****/#### *p* < 0.0001, ### *p* < 0.001, and ## *p* < 0.01, * *p* < 0.05 are considered significant references; NS, not significant.

RT-qPCR was measured to establish the impact of SANG on signaling pathways-mediated CCL2 release in TNBC cells. Initially, the obtained data indicated that CCL2 mRNAs expressions were reliably consistent with those of angiogenesis microarray and ELISA protein analyses (Figure 4). In both TNBC cell models under investigation, cells exhibited a significant response to TNF-α +/−SANG. TNF-α induced a significant upregulation in the mRNAs for CCL2 and its regulator gene IKBKE (*p* < 0.0001, Figure 4A,B). In MDA-MB-231 stimulated cells, SANG repressed the expression of these genes by 80% and 60%, respectively (*p* < 0.001–*p* < 0.0001, Figure 4A). For TNF-α-treated MDA-MB-468 cells, however, SANG caused a 60% inhibition in CCL2 mRNA (*p* < 0.01, Figure 4B) concurrent with 30% repression in the IKBKE gene (*p* < 0.001). Moreover, a minor but significant (*p* < 0.05) inhibition in the CCL2 gene was found in only SANG-treated TNBC cell models. The obtained PCR data confirmed our microarray data, which indicated a proportional relation between CCL2 and IKBKE expressions in TNBC cell models.

### 2.4. SANG Modulates Protein Expression of Signaling Pathways Mediating CCL2 Release in TNF-α-Stimulated TNBC Cells

**Figure 5:**
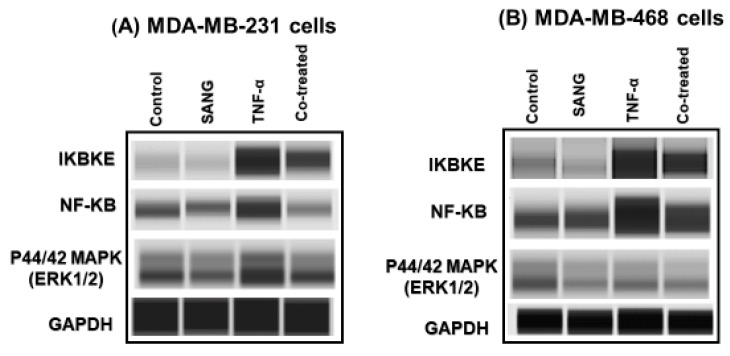

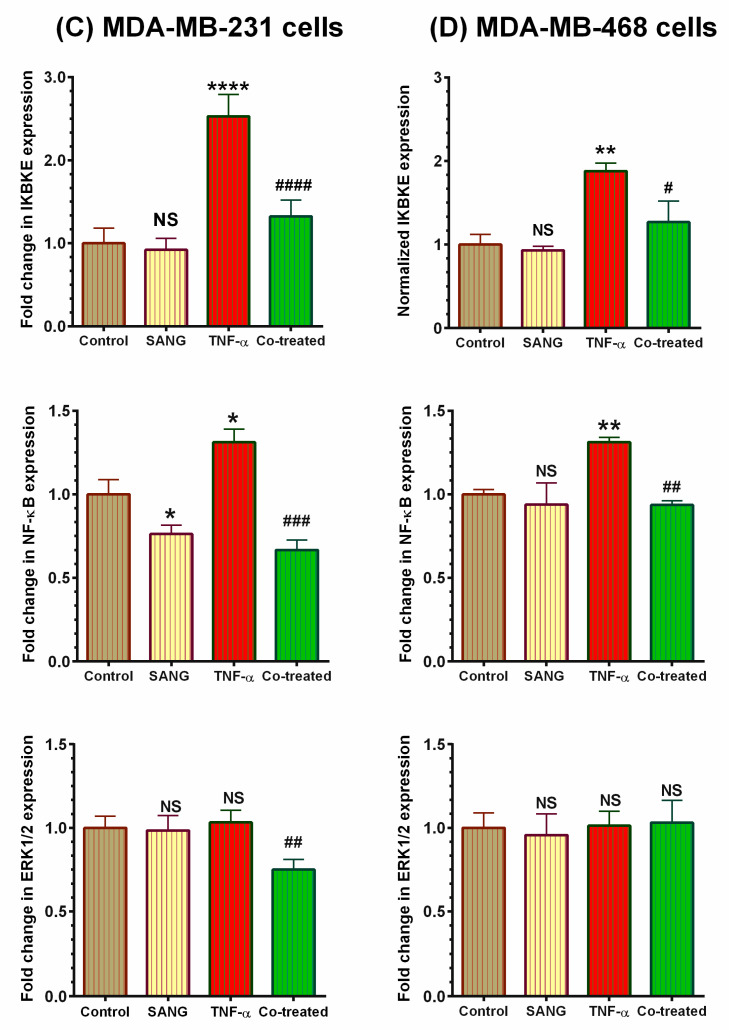
Effect of SANG on the expression of IKBKE, NF-κB, and ERK1/2 in TNF-α-stimulated TNBC cells. The expression of different proteins was measured in four samples for each experiment, corresponding to the resting, SANG-treated (2.0 µM in MDA-MB-231 cells and 1.5 µM in MDA-MB-468 cells), 50 ng/mL TNF-α-stimulated, and co-treated cells (TNF-α + SANG). The automated, simple Western system with its Compass software was used for band immunodetection for MDA-MB-231 (**A**) and MDA-MB-468 (**B**) cells. Quantitative analysis of GAPDH-normalized data for MDA-MB-231 (**C**) and MDA-MB-468 (**D**) cells. The data generated from two independent experiments (n = 4) are presented as the mean ± SEM. The significance of the difference between TNF α-treated cells vs. control cells (*) or between TNF-α-stimulated vs. co-treated cells (#) groups was analyzed using an unpaired *t*-test. */# *p* < 0.05, **/## *p* < 0.01, ### *p* < 0.001, and ****/#### *p* < 0.0001 are considered significant difference; NS, not significant.

We further investigated the implication of IKBKE protein in CCL2 inhibition and, consequentially, its impact on NF-κB-ERK1/2 signaling pathways in TNF-α-stimulated TNBC cells using the capillary electrophoresis Western analysis (Figure 5). The expressions of the three concerned proteins were measured in the sample lysates of four groups, control, SANG-treated, and TNF-α +/−SANG treated cells. As demonstrated in both Western bands (Figure 5A,B) and Compass software data analyses (Figure 5C,D), TNF-α-treated TNBC cells exhibited highly significant upregulated levels of IKBKE and NF-κB (*p* < 0.05–0.0001), while ERK1/2 protein was unchanged. These two upregulated proteins were then inhibited by SANG in the co-treated samples (*p* < 0.05–0.0001). In particular MDA-MB-231 cells, the co-existence of TNF-α and SANG conspicuously decreased IKBKE and NF-κB by 50% (*p* < 0.001–0.0001, Figure 5C). Moreover, ERK1/2 was significantly inhibited by 30% (*p* < 0.01). In comparison, the MDA-MB-468 model exhibited a lower inhibition of IKBKE and NF-κB (less than 30%, Figure 5D), while ERK1/2 protein expression was non-significant. These results may rationalize our previous results and indicate the possible anti-inflammatory-related antiangiogenic effects of SANG through inhibiting cytokine CCL2, surviving proteins of IKBKE, NF-κB, and ERK1/2, and their consequent signaling pathways.

### 2.5. SANG Inhibits Migration in TNBC Cells

**Figure 6:**
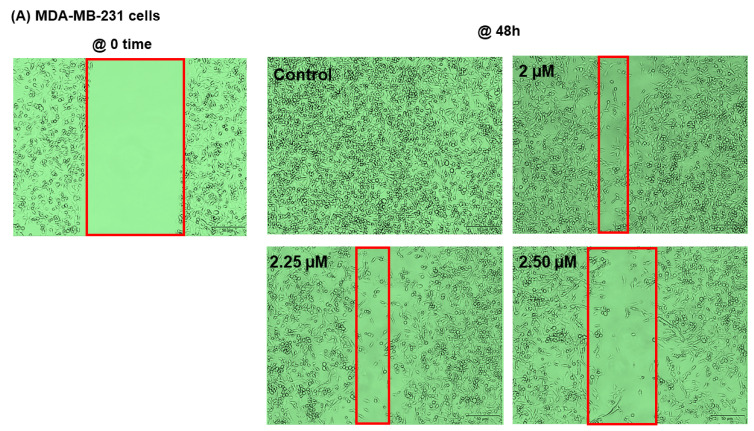

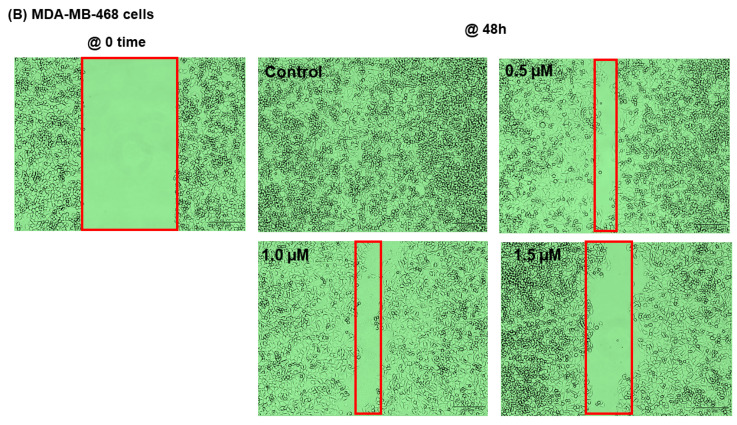

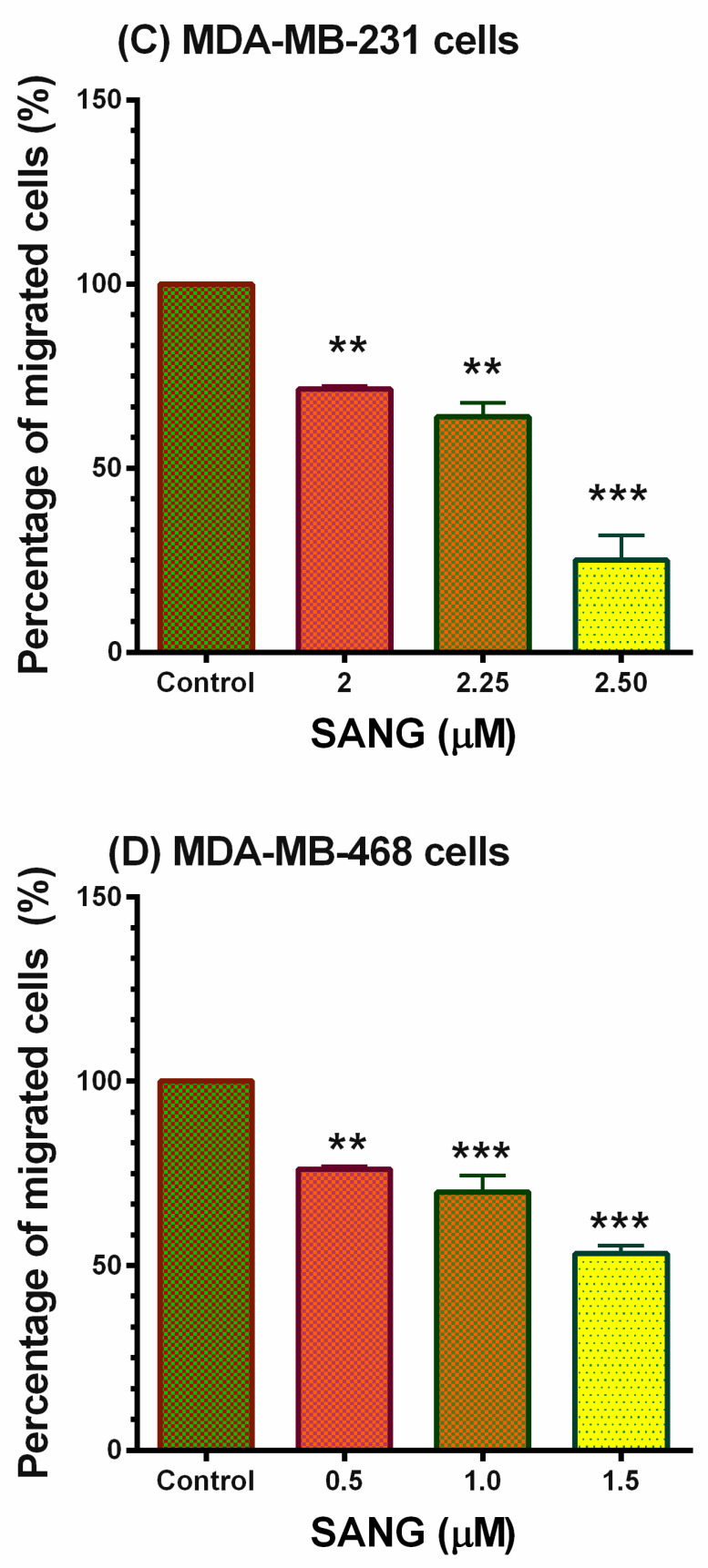
The effect of SANG on TNBC cells migration assay. TNBC cells of the same densities were seeded in two-well inserts and incubated overnight. Following the silicon insert removal and PBS wash, cells were treated with SANG and imaged at 0 time. Treated cells were incubated until the control gaps were closed. At 48 h, cells were again imaged (phase contrast, 20×), and the gap widths were analyzed. The red lines represent the approximate cell front (**A**,**B**). The generated data (**C**,**D**) are presented as the mean ± SEM of ≥2 studies (n = 3). One-way ANOVA, followed by Bonferroni multiple comparisons test, were performed to confirm the statistically significant anti-migration. ** *p* < 0.01 and *** *p* < 0.001 are considered significant differences. Scale bar: 50 μm.

Angiogenesis and metastasis are tightly linked mechanisms that advocate tumor progression at a distant site [66,67]. A migration assay was performed to delineate the potential antimetastatic effect of SANG (Figure 6). In establishing this investigation, we used SANG concentrations that allow for ≥75% viability (data not presented). At 48 h experimental period, the insert gap was sealed entirely in the control wells of both cell lines (Figure 6A,B). As depicted in the images, a proportional relationship was observed between the gap width and SANG concentrations in both cells under investigation. Consequently, a quantified significant inverse relationship was measured between the percentage of migrated cells and the tested concentrations. For comparison, a 75% inhibition (*p* < 0.001) in the migrated MDA-MB-231 cells was found at 2.5 µM SANG. Meanwhile, their counterpart MDA-MB-468 cells showed higher resistance to SANG with as low as 50% inhibition in cell migration (*p* < 0.001) at 1.5 µM SANG. The results obtained may suggest that SANG could have higher antimetastatic effects in MDA-MB-231 than in MDA-MB-468 cells, which is consistent with our antiangiogenic results.

## 3. Discussion

Abnormal angiogenesis is the lead mechanism in tumor growth and metastasis in many types of cancer, including BC [2,26,27,33,74,75,76,77]. Tumor growth associated with angiogenesis has been studied extensively in numerous in vivo models. To date, tumor angiogenesis continues to be explored, offering intriguing avenues of prognostic and therapeutic potential [23,25,78,79,80]. Many tyrosine kinase inhibitors and antibodies that target pro-angiogenic signaling have been approved for use in cancer therapy. Even though FDA-approved medications are growing rapidly, anti-angiogenic therapy has had limited success to date [3]. Therefore, novel therapeutic drugs and combinations that constrain angiogenesis with different approaches to enhance antitumor drugs will certainly improve patient survival [48].

The present study demonstrated the antiangiogenic mechanism of the natural alkaloid SANG in two models of TNF-α-stimulated TNBC cell lines. Initially, our data indicated that the co-existence of SANG and TNF-α exhibit almost comparable cytotoxic effects. Previous studies have suggested the crucial role of IKBKE in regulating the expression of CCL2 [81,82,83]. Subsequently, we highlighted the antiangiogenic and antimetastatic potentials of SANG by inhibiting the cytokine CCL2 along with its common regulator, IKBKE. These proteins enhance angiogenesis and metastasis by triggering critical signaling pathways, such as NF-κB and ERK1/2 [84,85], which were also inhibited by SANG, as revealed in our protein expression studies.

Cancer-associated inflammation and pathological angiogenesis share molecules and signaling pathways [18]. The cytokine TNF-α is highly upregulated in several inflammatory diseases. It can trigger various signaling pathways to provoke inflammation and enhance cell growth [35]. As an angiogenic factor, TNF-α is released by many normal and cancer cells to regulate thriving or pathogenic angiogenesis once binding to its ligand on the cell membrane [86,87]. Recent studies have proved its pro-angiogenic activity by activating various signaling pathways, such as NF-κB and ERK pathways [89,90]. Nevertheless, its exact function is usually associated with its pathological expression in tumor tissues [22]. In human BC, upregulated TNF-α promoted the proliferation of the mammary gland cell line T47D [90,91] and triggered several genes mediating cancer cells proliferation, invasion, and metastasis [92]. More importantly, increased TNF-α production may also release many important chemokines that significantly contribute to tumor angiogenesis [22], either directly or indirectly. In this paper, for method validation, stimulating both TNBC cell lines with TNF-α enhanced the expression of the CCL2 cytokine (Figure 1), a foundational finding that was previously reported by our lab and others [93,94,95].

In our investigation, the presence of a low concentration of SANG (2 µM max) dramatically downregulated the expression of CCL2 in TNF-α-activated MDA-MB-231 cells. That renders SANG more effective in this cell line than in MDA-MB-468 cells by more than 4.5-fold (Figure 2 and Figure 3). These results are generally consistent with SANG antiangiogenic effects in other cell lines at ≤ 2.5 μM [61].

In the context of cancer, CCL2, also known as monocyte chemoattractant protein-1 (MCP-1) [96], is the most cancer-dominant member of the CC-chemokine family [97]. CCL2 was found to play a considerable role as an angiogenic factor, comparable to the typical angiogenic vascular endothelial growth factor (VEGF)-A121 [98]. In MDA-MB-231 TNBC patients, the upregulated expression of CCL2 promoted tumorigenesis, metastasis, and decreased survival rate [99,100]. The administration of CCL2 also increased inflammatory angiogenesis [99,101]. In contrast, inhibiting this cytokine led to reduced metastasis and tumor aggressiveness [102]. Additionally, targeting CCL2 expression through genetic mutation or antibody administration reduced metastasis and enhanced prognosis [103,104]. Indeed, these findings hold promise in managing TNBC disease [99]. Therefore, the profound inhibition of CCL2 in MDA-MB-231 cells elucidate the notion that SANG could be a potential antiangiogenic agent for a subgroup of TNBC patients.

Our qRT-PCR gene expression profiling indicates that the mRNA expression of *CCL2* and *IKBKE* was significantly upregulated by TNF-α, followed by a significant inhibition by SANG (Figure 4). The gene *IKBKE* (also known as IKKε) codes for IKBKE and is a crucial kinase of the IKK family and the main regulator of CCL2 protein [105]. Indeed, these altered genes are implicated in different signaling pathways, including NF-κB and ERK, and promote the upregulation of the CCL2 cytokine [84,85,106]. In agreement with our findings, a recent study revealed that the cytokine TNF-α upregulates *CCL2* expression [107] and its regulator *IKBKE* gene [108]. Additionally, the simultaneous overexpression of *CCL2* and *IKBKE* has been established by many studies [81,82,94]. In TNBC cells, the *IKBKE* oncogene is overexpressed in 60% of BC tissues [85,109]. In addition to its main function supporting BC cell viability, upregulated *IKBKE* increased resistance to antineoplastic therapy by eluding tamoxifen-promoted apoptosis [110,111]. In contrast, *IKBKE* knockdown in human BC cells [112], including TNBC [113], was accompanied by a reduction in cell viability, proliferation rate, migration, and invasion, and ultimately led to cell death. In MDA-MB-231 cells, *CCL2* gene silencing led to inhibition of CCL2 expression, which—in turn—led to the significant inhibition of tumor growth, cell proliferation, and increased necrosis [99,114]. Hence, the implication of *IKBKE* in breast carcinoma became a promising prospect for targeted therapies [109]. The current findings corroborate earlier research and suggest *IKBKE* repression as the anticipated mechanism of SANG-mediated *CCL2* suppression [108].

In the cancer microenvironment, the manipulation of crucial signaling pathways, such as NF-κB and ERK/MAPK, is considered a promising target for cancer therapies. In our Western analysis, SANG significantly inhibited the protein expression of IKBKE and NF-κB signaling pathways in both TNF-α-stimulated TNBC cell models (Figure 5).

The transcription factor, the nuclear factor-kappa B (NF-κB), has been proven to promote inflammatory angiogenic pathways and boost tumor growth through neovascularization [115]. The protein under investigation, NF-κB p65, is the most common activated form in the NF-κB family [116]. As a critical activator in inflammation and cancer, NF-κB controls the release of TNF-α, CCL-2, various inflammatory factors, and tumor-related genes [82]. These activities were demonstrated to influence tumor development, invasion, and metastasis [117]. Interestingly, the released TNF-α enhances NF-κB signaling pathway, also known as auto-stimulation [104]. Many pharmaceutical and drug discovery research emphasized the value of targeting the NF-κB p65 signaling pathway [117]. On the other hand, IKBKE plays an essential role in activating the NF-κB signaling pathway, among others [106,119,120]. IKBKE is a typical upstream regulator of the transcription factor NF-κB pathway, which has been significantly involved in the pathogenesis of TNBC [76,77,78,79]. Consequently, silencing IKBKE has been reported to reduce the NF-κB activity and inhibit proliferation, clonogenicity, angiogenesis, migration, invasion, and metastasis in BC cells and many other cancer types [117,121]. Thus, the potential of SANG to inhibit IKBKE and NF-κB signaling is a promising compound to control TNBC growth and metastasis.

The concept that CCL2 promoted the survival and migration of MDA-MD-231 cells by activating ERK1/2 signaling encouraged us to emphasize this mechanism [114]. In TNF-α-stimulated MDA-MB-231 cells, SANG exclusively inhibited ERK1/2 expression compared to the MDA-MB-468 model (Figure 5). In addition to IKBKE, the ERK1/2 signaling pathway plays a considerable role in activating NF-κB [122,123]. Parallel to NF-κB, upregulated ERK (also known as a mitogen-activated protein kinase, MAPK) was correlated with the low survival rate in TNBC patients [124,125]. In MDA-MB-231 TNBC cells, upregulated ERK pathway was implicated in uncontrolled proliferation, migration, and cancer cell invasion [84,126], concurrent with resistance to apoptosis [127,128,129]. Indeed, ERK signaling enhances cell survival, proliferation, and migration through signals transduction and release of cytokines and growth factors [114]. The concept that IKBKE and CCL2-CCR2 signaling activate the downstream signaling of ERK pathways [108,122] promoted intense investigations. Therefore, targeting this signaling by SANG, specifically in MDA-MB-231, could be a prospective strategy for managing this cell line.

Moreover, CCL2 and its CCR2 receptor have been revealed to play pivotal roles in cancer metastasis’s early and late stages [130,131,132]. The role of CCL2 in metastasis is established by supporting survival, proliferation, inflammatory angiogenesis, and increasing cancer cell migration and invasion [130,133]. Certainly, CCL2 is involved in MMP14 upregulation, necessary for neovascularization and endothelial cell migration [43,47]. In various models of angiogenesis, CCL2-CCR2 signaling was demonstrated as a mediator of neovascularization, hence sustaining cell proliferation and viability, promoting cancer cell invasion and migration, and provoking inflammation and angiogenesis [43,98,130,134]. In contrast, CCL2 deficiency reduced macrophage recruitment and angiogenesis [135], leading to a reduction in metastases. Therefore, our finding that the SANG can decrease CCL2 expression may contribute, as a result, to inhibiting metastasis in TNBC [44].

We further evaluated the compound’s capacity to halt migration in non-stimulated cells. Our migration analysis suggested the antimetastatic activity of SANG in both cell lines under investigation with relatively higher effects in MDA-MB-231 cells (Figure 6). Angiogenesis is cohesively linked to metastasis, fostering tumor progression at distant sites [136,137]. Metastasis is the key to cancer-related death [138] since, in the metastasis process, tumor cells segregate from the initial tumor and invade surrounding or distant tissues to establish a secondary tumor [48]. Patients with BC are at risk for metastasis during their lives [139]. Metastatic BCs, also known as stage IV or advanced BCs, can be found in lymph nodes in the armpit and/or in distant sites, such as the lung, liver, bones, and brain [140]. Even after the primary tumor is removed, microscopic tumor cells or micro-metastases may remain in the body, allowing cancer to return and disseminate [141]. It is estimated that between 25% and 50% of BC patients may develop more spread metastases after diagnosis. Unfortunately, patients with metastatic disease have a poor prognosis, with a 5-year survival rate [138]. Therefore, our finding that the natural alkaloid SANG can inhibit metastasis is worthy of consideration [44]. However, more studies are needed to investigate the antiangiogenic effect of SANG in TNBC in vivo models.

## 4. Materials and Methods

### 4.1. Cell Culture and Media

The TNBC cell lines used in this study, MDA-MB-231 (ATCC^®^ HTB-26™) and MDA-MB-468 (ATCC^®^ HTB-132™), were purchased from ATCC (Manassas, VA, USA). These cell lines MDA-MB-231 and MDA-MB-468 were isolated and immortalized from Caucasian American (CA) and African American (AA) breast tumors, respectively. Cell culture media and the required supplements were purchased from different vendors, such as VWR International (Radnor, PA, USA), ATCC, Thermo USA Scientific (Ocala, FL, USA), and Santa Cruz Biotechnology Inc. (Dallas, TX, USA). These cells were used as monolayers in tissue culture (TC) flasks and incubated under a controlled environment (37 °C and 5% CO_2_ humidified incubator). The TNBC cells were cultured according to the previously mentioned guideline [142]. Briefly, we used Dulbecco’s Modified Eagle Medium (DMEM), supplemented with 4 mM L-glutamine, 10% heat-inactivated fetal bovine serum (FBS), a100 U/mL penicillin, and 0.1 mg/mL streptomycin (1% P-S). Media were replaced with a fresh one after washing the cells with Dulbecco’s phosphate-buffered saline (DPBS). The confluent flasks were sub-cultured with trypsin/ethylenediaminetetraacetic acid (EDTA, 0.25%). The phenol-free experimental media were supplemented identically to cell culture one, except heat-inactivated FBS that was lower (2.5%) in all conducted assays, except the cell migration study.

### 4.2. Cell Viability Assay

This experiment was designed to determine the combined effect of TNF-α and SANG on cell viability using Alamar Blue^®^ (AB^®^, Sigma-Aldrich, St. Louis, MO, USA), as previously mentioned [94]. The tested alkaloid compound sanguinarine (SANG) in chloride hydrate form of ≥98% (HPLC) was purchased from Sigma-Aldrich and reconstituted in dimethyl sulfoxide (DMSO, ATCC) at a final stock concentration of 15 mM, aliquoted and stored in −20 freezers. The cells under investigation were seeded into 96-well plates at a density of 5 × 10^4^ cells/100 µL/well and incubated overnight in the adjusted cell culture incubator. Cells were then treated in another 100 µL of experimental media containing either DMSO (≤0.1%) for the control or working solution of SANG (0–5 µM) and 50 ng/mL of tumor necrosis factor-α (TNF-α, Ray Biotech, Norcross, GA, USA). After 24 h, the freshly prepared AB^®^ resazurin solution (0.5 mg/mL in sterile cell culture water) was added to each well at a concentration 10%, and the plates were returned to the cell culture incubator for a further 4 h. A Synergy HTX Multi-Mode microplate reader (BioTek Instruments, Inc., Winooski, VT, USA) was used for measuring the cell viability at excitation/emission of 530/590 nm. Cell viability analysis was established using the average of three independent experiments.

### 4.3. Human Angiogenesis Array

Human angiogenesis microarray (Ray Biotech, AAH-ANG-1000) was used to measure the expression of cytokine-mediated angiogenesis in TNBC-treated cells. For each cell line, we employed a set of four 75-cm^2^ TC flasks seeded with 10 × 10^6^ cells/each. Each set of flasks included control, SANG-treated, TNF-α-stimulated, or co-treated (TNF-α + SANG). The chosen concentrations for this preliminary study were designed based on the data of the cytotoxicity assay with a mild impact on cell viability. Accordingly, control cells were treated only with the highest used DMSO (≤0.1%) and SANG-treated cells were exposed to either 2 µM or 1.5 µM in MDA-MB-231 and MDA-MB-468 cells, respectively. In the third flask set, each cell line was equally treated with 50 ng/mL of TNF-α. Meanwhile, the co-treated cells were exposed to a cocktail of 50 ng/mL of TNF-α and SANG at 2 µM for MDA-MB-231 cells or 1.5 µM for MDA-MB-468 cells. Following the 24 h exposure period, cells were mechanically collected in a fresh set of falcon tubes and centrifuged. For each sample, the cell-free supernatant and the corresponding cell pellet were stored at −80 °C for further studies. A semiquantitative assay—using antibody-coated array blots—was used to measure the expression of different cytokines-mediating angiogenesis in the previously collected cell-free supernatant. Initially, the four membranes presenting each set were placed carefully in the assigned tray without touching the antibody-coated surface. All membranes were equally blocked with 2 mL of the provided buffer. The blocking buffer was then aspirated, and 1 mL of freshly thawed cell-free supernatant was pipetted to the corresponding blot. The tray was kept overnight at 4 °C with brief shaking. After that, supernatants were aspirated from each chamber, and the membranes were washed using the kit buffers. Next, all membranes were incubated for 2 h at RT with 1 mL of freshly diluted biotinylated antibody, followed by a second wash. The membranes were lately exposed for an additional 2 h to the fresh constituted horseradish peroxidase-conjugated streptavidin (HRP-Streptavidin) and followed by final washes. An assigned chemiluminescence buffer was used to detect the cytokine intensities on the different blots. The blot images were depicted within 5 min using a Flour-S Max Multi-imager (Bio-Rad Laboratories, Hercules, CA, USA). Quantifying the spots intensities were established with the Quantity One Software (Bio-Rad Laboratories, Hercules, CA, USA). Excel-based data analysis software for Human Angiogenesis Array AAH-ANG-1000 was used for normalizing the cytokine intensities.

### 4.4. Enzyme-Linked Immunosorbent Assay (ELISA)

To measure the protein level (pg/mL) of the chemokine C-C Motif Ligand 2 (CCL2, also known as human monocyte chemoattractant protein-1, MCP-1), we used an ELISA kit (Ray Biotech, ELH-MCP1) for this particular cytokine. Briefly, the standard curve for CCL2 was prepared side-by-side with supernatant representing different treatments. In a time-controlled sequence, the antibody pre-coated 96-well ELISA microplates were exposed to the following: a 100 µL of each sample/standard for 2.5 h, a 100 µL freshly diluted biotinylated antibody for an hour, a 100 µL Streptavidin solution for 45 min, and a 100 µL of the substrate reagent. Lastly, 50 µL of a stop-solution was added to each well to terminate the reaction. The intensity of CCL2 in the standard and each sample was measured at 450 nm using the Synergy HTX Multi-Reader (BioTek). The assay was repeated 3 times.

### 4.5. Gene Expression Study

#### 4.5.1. RNA Extraction

This assay extracted the total RNA from the −80 °C-frozen cell pellets, as mentioned above. All samples were initially homogenized with 1 mL of Trizol^®^ (Thermo Fisher Scientific, Inc., Waltham, MA, USA) before adding 200 µL of chloroform (Sigma Aldrich, St. Louis, MO, USA). Samples were then vortexed and centrifuged for 15 min at 10,000× *g* and 8 °C. The upper RNA-rich layer was transferred to a clean tube and mixed with 500 µL of 2-propanol to extract RNA pellets. Following 20 min centrifugation at 10,000× *g*, the liquid part of all tubes was aspirated, and the extracted pellets were then washed with 75% ethanol, air-dried, and reconstituted in nuclease-free water.

#### 4.5.2. Complementary DNA (cDNA) Synthesis

To synthesize cDNA, we first determined the concentration of the reconstituted RNA in each sample using a Nanodrop spectrophotometer (Thermo Fisher Scientific, Inc., Waltham, MA, USA). Then, RNA at a concentration of 200 µg/mL combined with a 1X DNase cocktail (DNA-free™ kit, Thermo Fisher Scientific) was incubated for 30 min at 37 °C. The reaction was terminated by adding a DNase inactivator. After that, all samples were centrifuged at 9000 rpm for 3 min to collect the DNA-free supernatant. To obtain cDNA, we followed the protocol provided with the iScript™ cDNA Synthesis kit (Bio-Rad Laboratories, Hercules, CA, USA). For a final volume of 20 µL, each well of the 96-well PCR plates was loaded with 9 µL of nuclease-free water, 5 µL of the DNA-free supernatant, and 6 µL of advanced reaction mix reverse transcriptase (RT) cocktail. The RT reaction was initiated at 46 °C for 20 min, followed by inactivation at 95 °C for 1 min, using the CFX96 Touch Real-Time PCR Detection System (Bio-Rad). Instantly, the obtained cDNA plates were placed in a −80 °C freezer.

#### 4.5.3. Quantitative Reverse Transcription-Polymerase Chain Reaction (qRT-PCR)

The expression of *CCL2* and—its main regulator—an inhibitor of nuclear factor-kappa B kinase subunit epsilon (*IKBKE*) was measured using the CFX9 Real-Time System (Bio-Rad Laboratories, Inc.). Briefly, 50 ng/sample of the previously synthesized cDNA was combined with 2× real-time Master Mix solution in a final volume of 20 µL. The PCR cycle started with 2 min incubation at 95 °C, followed by 39 cycles of amplification. Each cycle included denaturation at 95 °C for 10 s, annealing at 60 °C for 30 s, and finally, melting curve at 65–95 °C for 5 s. The primers used in this PCR assay were compatible with the genes under investigation, as summarized in Table 1. The mRNA regulation for the examined genes was normalized using GAPDH as a reference gene. All analyzed data were generated from three independent studies for each primer.

### 4.6. Capillary Electrophoresis Western Analysis

This study was established using Wes Fully Automated Western System ver.6.0 (Protein Simple, San Jose, CA, USA). Cell setup and treatment were designed identical to the human angiogenesis assay (Section 2.3). For each sample, the cell pellet was lysated using a cocktail of lysis buffer and protease inhibitor. Protein quantification in cell lysates was measured using the protocol of the Pierce^TM^ BCA protein assay kit (Thermoscientific, Rockford, IL, USA). For band immunodetection, the primary antibodies of IKBKE (2690S), NF-κB p65 (8242S), extracellular signal-regulated kinase (ERK1/2, also known as p44/42 MAPK) (9102 S), and GAPDH (14C10) were used. Rabbit mAb (2118S) was used as the endogenous control antibody, and anti-rabbit IgG, HRP-linked antibody (7074S), was used as the secondary antibody. Both primary and secondary antibodies were purchased from Cell Signaling Technology (Beverly, MA, USA). The protein expression in each sample was determined using a 12–230 kDa Wes Separation Module-25 capillary cartridge and its compatible Anti-Rabbit Detection Module kit. According to the protocol of Protein Simple, optimum concentrations for the cell lysates and antibodies under investigation were established. For both cell models, the applied concentration of the cell lysates was 0.5 mg/mL. For MDA-MB-231 samples, the dilution factor for the three antibodies under investigation was 1:100. Meanwhile, the dilution factor of 1:10 for IKBKE and NF-κB and 1:50 for ERK1/2 were found ideal for examining their expression in different samples generated from MDA-MB-468 cells. We normalized the generated data using the endogenous control GAPDH. The output data were generated from two independent experiments (n = 4) and analyzed using Protein Simple Compass software and Prism-GraphPad software ver. 6.2 (San Diego, CA, USA).

### 4.7. Migration Assay

This assay was established using two well-self-insertion kits (Ibidi^®^, VWR). Each well of the 6-well plate was inserted with the 2-well silicone insert. MDA-MB-231 and MDA-MB-468 cells were seeded overnight at 3.5 × 10^4^/70 µL media/compartment. In sequence, the inserts were carefully removed, the media with floating cells were decanted, and all wells were washed twice with PBS. Cells were then treated with low concentrations of SANG (0–2.5 µM in MDA-MB-231 cells and 0–1.5 µM in MDA-MB-468 cells). Figures were first captured at 0 times, and all plates were placed in the cell culture incubator until the gap of the control wells was totally closed. At 48 h exposure period, figures depicting different concentrations were captured, and the gap width for each well was measured. The assay was repeated three times with n = 3 for each treatment group. The % of migrated cells was calculated using the following equation

$$\frac{A2}{A1}\left(Treated\;cells\right)-\frac{A2}{A1}\left(Control\;cells\right)\times 100$$

*A*2; the gap width after 48 h, *A*1; the gap width at 0 time.

### 4.8. Statistical Analysis

The data of the present study are expressed as the means ± standard error of the mean (SEM). Prism-GraphPad software analysis generated the IC_50_ values by nonlinear regression model of log (inhibitor) vs. normalized response-variable slope with the R^2^ best fit and the lowest 95% confidence interval. An unpaired *t*-test was used to compare the two groups. Comparisons of more than two groups were determined using one-way analysis of variance (ANOVA) followed by Bonferroni post-test. In all analyses, *p* < 0.05 indicates a statistical significance difference. A detailed data analysis was performed, as mentioned in each figure’s caption.

## 5. Conclusions

The current investigation revealed the mechanism underlying the antiangiogenic potential of the natural alkaloid compound sanguinarine (SANG) in TNF-α-stimulated TNBC models, MDA-MB-231, and MDA-MB-468 cells. Our findings indicate that SANG potently inhibited the cytokine CCL2 in MDA-MB-231 with less potency in MDA-MB-468. Additionally, SANG inhibited the CCL2 regulator gene IKBKE, NF-κB, and ERK1/2 signaling pathway. The inhibition of ERK1/2 total protein in MDA-MB-231 cells could rationalize the potential higher antimetastatic effect. Further research on SANG is needed to assess the impact of SANG on the total and phosphorylated protein levels of other ERK family members. Indeed, more studies are needed to investigate the antiangiogenic effect of SANG in TNBC in vivo models. Therefore, our data show that SANG is a potent inhibitor of CCL2 and its related pathways. With its effective antiangiogenic mechanism, SANG has a promising potential to develop a therapy for TNBC disease.

## Figures and Tables

**Table 1:** List of primers utilized in studying gene expression alteration in PCR experiments.

Primer Name	GenBank Accession No.	Amplicon Context Sequence
*MCP1/CCL2*	NM_002982.4	ACTGAAGCTCGCACTCTCGCCTCCAGCATGAAAGTCTCTGCCGCCCTTCTGTGCCTGCTGCTCATAGCAGCCACCTTCATTCCCCAAGGGCTCGCTCAGCCAGATGCAATCAATGCCCCAGTCACCTGCTGTTATAACTTCACCAATAGGAAGATCTCAGTGCAGAGGCTCGCGAGCTAT
*IKBKE*	NM_014002.4	GGCTTGGCTACAACGAGGAGCAGATTCACAAGCTGGATAAGGTGAATTTCAGTCATTTAGCCAAAAGACTCCTGCAGGTGTTCCAGGAGGAGTGCGTGCAGAAGTATCAAGCGTCCTTAGTCACACACGGCAAGAGGATGAGGGTGGTGCACGAG
*GAPDH*	NM_002046.7	GTATGACAACGAATTTGGCTACAGCAACAGGGTGGTGGACCTCATGGCCCACATGGCCTCCAAGGAGTAAGACCCCTGGACCACCAGCCCCAGCAAGAGCACAAGAGGAAGAGAGAGACCCTCACTGCTGGGGAGTCCCTGCCACAC

## Data Availability

Not applicable.
